# Learning to Navigate in Chemical Fields Without A Map at Low Reynolds Numbers

**DOI:** 10.1002/advs.202510092

**Published:** 2025-08-28

**Authors:** Yangzhe Liu, On Shun Pak, Alan C. H. Tsang

**Affiliations:** ^1^ Department of Mechanical Engineering The University of Hong Kong Pokfulam Road Hong Kong China; ^2^ Department of Mechanical Engineering and Department of Applied Mathematics Santa Clara University Santa Clara CA 95053 USA

**Keywords:** chemotaxis, microswimmers, navigation, reinforcement learning

## Abstract

Effective navigation of microswimmers relies on their ability to search for unknown target locations using limited information provided by local environmental cues. Biological microswimmers have evolved versatile strategies to achieve such mapless navigation. Yet, achieving autonomous navigation in artificial microswimmers, comparable to that of their biological counterparts, remains a significant challenge. In this work, deep reinforcement learning is employed to equip a reconfigurable artificial microswimmer with the ability to navigate and search for a target without relying on a pre‐existing map. These results demonstrate how this mapless swimmer can effectively navigate toward a chemical source by responding to local chemical signals. Remarkably, the swimmer adapts its locomotory gaits in response to local chemical fields, exhibiting a run‐and‐tumble strategy reminiscent of bacterial chemotaxis. Unlike map‐based swimmers, which depend on pre‐existing maps for successful navigation, the mapless swimmer achieves robust performance even in chemical fields that significantly deviate from its training environment, including time‐varying fluctuating environment. Moreover, the swimmer can progressively explore complex chemical fields with multiple local maxima, effectively searching for regions of higher concentration. These findings present a promising approach toward achieving autonomous navigation for artificial microswimmers in unknown environments.

## Graphical abstract

Deep reinforcement learning enables a reconfigurable artificial microswimmer to exhibit run‐and‐tumble strategy to navigate toward a chemical source. This mapless microswimmer can robustly navigate in diverse chemical fields without relying on a pre‐existing map.

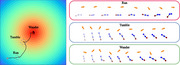

## 1 Introduction

For microorganisms, survival in complex and dynamic environments hinges on two critical capabilities: the ability to move and the ability to navigate effectively. Self‐propulsion is essential for microorganisms to explore their surroundings, locate nutrients, and escape from harmful conditions. Microorganisms have evolved a variety of self‐propulsion mechanisms to adapt to the physical constraints of their microscopic world, where viscous forces dominate over inertial forces.^[^ ^1^, ^2^ ^]^ Some bacteria, for example, use rotating flagella to propel themselves through fluids,^[^ ^3^, ^4^ ^]^ while certain eukaryotic cells achieve movement by beating their flagella or cilia in coordinated manners.^[^ ^5^, ^6^ ^]^ These mechanisms enable microorganisms to traverse their environments with remarkable agility. Such self‐propulsion mechanisms designed by nature have inspired significant efforts in the development of artificial microswimmers – tiny robots capable of autonomous movement at the microscale.^[^ ^7^, ^8^, ^9^ ^]^ These artificial systems hold great promise for biomedical applications, including microsurgery and targeted drug delivery.^[^ ^10^, ^11^, ^12^, ^13^ ^]^ While some designs directly mimic the propulsion strategies observed in nature, others employ novel physical or physico‐chemical mechanisms to overcome the challenges of achieving self‐propulsion in the microscopic realm.^[^ ^14^, ^15^, ^16^ ^]^

Movement alone, however, is not sufficient for survival. Equally vital is the ability to navigate, which involves responding to environmental cues such as chemical gradients and light to guide microorganisms toward favorable conditions and away from threats.^[^ ^17^, ^18^, ^19^, ^20^, ^21^ ^]^ Microorganisms have evolved different tactic behaviors that enable them to detect and respond to local cues, allowing them to navigate effectively even in complex or unpredictable environments. Chemotaxis, for example, is a critical tactic behavior that enables microorganisms to sense and move along chemical gradients. To achieve this, chemotactic cells often modulate their body shapes or locomotory gaits,^[^ ^22^, ^23^, ^24^, ^25^ ^]^ adapting their movements to explore their environment and navigate towards nutrient‐rich areas or away from harmful substances. This behavior is particularly well‐documented in bacteria like *Escherichia coli*, which utilize a run‐and‐tumble strategy – alternating between smooth swimming and abrupt directional changes – to successfully perform chemotaxis. These sophisticated behaviors of biological cells have inspired the development of artificial microswimmers, with the goal of equipping them with the autonomous capabilities observed in nature, enabling them to perform complex biomedical tasks.^[^ ^26^, ^27^, ^28^, ^29^ ^]^ However, designing artificial systems that can autonomously move and navigate like their biological counterparts, without relying on pre‐programmed paths or complete knowledge of the environment, remains a significant challenge.

Recently, machine learning has emerged as a powerful approach for designing artificial intelligent systems capable of performing complex tasks without explicit programming.^[^ ^30^, ^31^, ^32^, ^33^ ^]^ In the context of microswimmers, pioneering research has leveraged different machine learning techniques to develop effective locomotory gaits for self‐propulsion and navigation at the microscopic scale.^[^ ^34^, ^35^, ^36^, ^37^, ^38^, ^39^, ^40^, ^41^, ^42^, ^43^, ^44^, ^45^, ^46^ ^]^ In particular, recent work has demonstrated that artificial microswimmers can learn chemotaxis – autonomously adapting their shape to move toward regions of higher field concentrations in one dimension – using genetic algorithms.^[^ ^47^ ^]^ Although limited to a single dimension, this study represents a significant advancement, demonstrating how machine learning‐driven decision‐making can enable chemotaxis in artificial microswimmers. In our work, we take this a step further by employing a deep reinforcement learning (RL) approach to achieve mapless navigation of artificial microswimmers in diverse chemical landscapes within a higher‐dimensional environment. Mapless navigation refers to the ability to navigate an environment without relying on a pre‐existing map or detailed knowledge of the terrain, making it particularly useful in dynamic or unknown settings where creating and maintaining an accurate map is challenging or impractical. Our work explores the chemotactic behaviors that emerge in this mapless context and evaluates the robustness of the RL‐driven navigation strategies in complex chemical landscapes. The results pave the way to developing more intelligent artificial microswimmers capable of autonomous, adaptive navigation in complex environments.

## 2 Experimental Section

### 2.1 Reconfigurable Microswimmer Model

**Figure 1:**
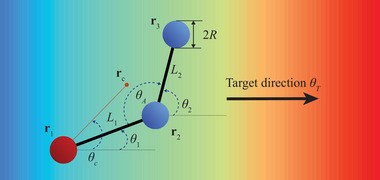
Schematic of the reconfigurable swimmer model. The swimmer comprises three spheres with radius *R* connected by two extensible links with lengths *L*_1_ and *L*_2_. The maximum length of the links is set to be *L*. The angles of the links are denoted as θ_1_ and θ_2_. The swimmers can extend or contract the angle between the two links θ_*A*_ within a range of $\frac{2}{3}\pi \leq \theta_{A}\leq \frac{4}{3}\pi$. The position for each sphere is denoted as **r**_*i*_(*i* = 1, 2, 3). The configuration of the swimmer is defined by the centroid $r_{c}=\sum_{i=1}^{3}r_{i}/3$ and orientation θ_*c*_. The swimmer can detect the localized chemical signal at its centroid to determine a target direction θ_*T*_ for navigation of the chemical source.

As a demonstration of the RL approach, a reconfigurable microswimmer model consisting of three rigid spheres of radius *R* connected by two extensible links of lengths *L*_1_ and *L*_2_ (**Figure** **1**) was considered. The configuration of the swimmer was represented by its centroid $r_{c}=\sum_{i=1}^{3}r_{i}/3$ and an orientation given by θ_*c*_ = *arg*(**r**_*c*_ − **r**_1_), where **r**_*i*_ are the positions of the spheres' centers (*i* = 1, 2, 3). This reconfigurable swimmer could be considered as a variant of the canonical three‐sphere Najifi‐Golestanian swimmer, Purcell's rotator and many others,^[^ ^14^, ^48^, ^49^, ^50^, ^51^, ^52^ ^]^ which could modulate the lengths (*L*_1_, *L*_2_) and the relative angle (θ_*A*_) of its two links to exhibit a rich variety of gaits for complex locomotion, thereby enabling it to self‐propel and reorient itself like biological swimmers.^[^ ^39^ ^]^

The hydrodynamics of the swimmer at low Reynolds numbers is governed by the Stokes equation

(1) $$\begin{array}{c} \mu \nabla^{2}u=\nabla p,\;\;\nabla \cdot u=0 \end{array}$$

where μ, **u**, and *p* denote the dynamic viscosity, velocity field, and pressure field of the fluid, respectively. In this Stokesian regime, the velocities of each sphere **V**_*i*_ and the hydrodynamic force **F**_*i*_ acting on them are linearly related as

(2) $$V_{i}=\sum_{j=1}^{N}H_{ij}F_{j}$$

where **H**_*ij*_ is the *Oseen‐Burger* tensor^[^ ^53^, ^54^, ^55^ ^]^ given by

(3) $$H_{ij}=\left\{\begin{array}{cc} I/6\pi \mu R, & i=j\\ \left(1/8\pi \mu |r_{ij}|\right)\left(I+r_{ij}r_{ij}/{\left|r_{ij}\right|}^{2}\right), & i\neq j \end{array}\right.$$

here **I** is an identity matrix and **r**_*ij*_ = **r**_*i*_ − **r**_*j*_ is the position vector joining spheres *i* and *j*. The relative positions between the spheres can be further expressed in terms of the unit vector ${\hat{r}}_{ij}$ between spheres *i* and *j* as

(4) $$\begin{array}{cc} r_{2}-r_{1} & =L_{1}{\hat{r}}_{21}\\ r_{3}-r_{2} & =L_{2}{\hat{r}}_{32} \end{array}$$

Therefore, the relative velocities between the spheres are given by

(5) $$\begin{array}{cc} V_{2}-V_{1} & ={\dot{L}}_{1}{\hat{r}}_{21}+L_{1}{\dot{\theta }}_{1}{\hat{r}}_{21}^{⊥}\\ V_{3}-V_{2} & ={\dot{L}}_{2}{\hat{r}}_{32}+L_{2}{\dot{\theta }}_{2}{\hat{r}}_{32}^{⊥} \end{array}$$

where ${\hat{r}}_{ij}^{⊥}$ is the perpendicular unit vector along the link, with $\partial_{t}{\hat{r}}_{ij}=\dot{\theta_{j}}{\hat{r}}_{ij}^{⊥}$. The swimmer can modulate the lengths of its links and relative angle at actuation rates, ${\dot{L}}_{i}$ and ${\dot{\theta }}_{A}$, respectively, to perform its locomotory gaits. Combining the above relations with the force‐free and torque‐free conditions,

(6) $$\begin{array}{c} \sum_{i=1}^{3}F_{i}=0,\;\;\;\sum_{i=1}^{3}T_{i}=0 \end{array}$$

the hydrodynamics of the microswimmer, as well as the relative positions and velocities of the spheres, are fully determined. We remark that the hydrodynamic description based on the *Oseen‐Burger* tensor was valid when the spheres are sufficiently far apart. Therefore, in this work, a constraint was enforced on the maximum contraction of the links (0.6*L* ⩽ *L*_1_, *L*_2_ ⩽ *L*) and the maximum relative orientation between the spheres ($\frac{2}{3}\pi \leq \theta_{A}\leq \frac{4}{3}\pi$) to avoid close proximity of the spheres. Hereafter, lengths were scaled by the maximum link length *L*, velocities by a characteristic actuation rate *V*_*c*_, and time by *L*/*V*_*c*_ when presenting the results, using the same symbols for the dimensionless quantities as for their dimensional counterparts for convenience.

### 2.2 Chemotaxis Via Deep RL

**Figure 2:**
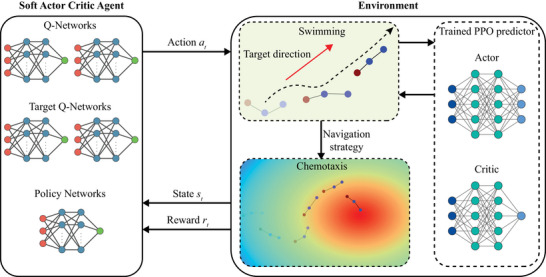
Schematic of the deep RL structure for learning chemotactic navigation. A two‐step learning scheme is implemented for the learning process for locomotion and navigation. In the environment (right), the swimmer already knows in prior how to adapt its locomotory gait to swim in a given targeted direction θ_*T*_ via a trained PPO predictor. The swimmer model passes the information about the current state and the reward to the Soft Actor Critic Agent. The SAC network then develops a policy and advises a new action for the swimmer in the next iteration to yield a navigation strategy for chemotaxis. All the neural networks contain two hidden layers, and each hidden layer has 128 neurons.

Successful chemotaxis required a microswimmer to not only identify the target direction toward the chemical source based on local chemical cues but also determine how to adapt its locomotory gaits to effectively re‐orient and self‐propel in that direction. However, simultaneously learning the chemotactic navigation strategy and the gait adaptation strategy was computationally intensive due to the complexity and large number of parameters involved in the learning process. To address this challenge, a two‐step learning scheme using deep RL was developed to divide the learning process into separate sessions for gait adaptation and chemotactic navigation (**Figure** **2**).

#### 2.2.1 Two‐Step Learning Scheme

In the first learning session, a Proximal Policy Optimization (PPO) algorithm was implemented to train a predictor for locomotory gaits using a deep neural network with an actor‐critic structure.^[^ ^56^ ^]^ The actor network advises the locomotory gaits required for a swimmer with a given configuration (*L*_1_, *L*_2_, θ_*A*_) to swim in a designated target direction θ_*T*_, whereas the critic network evaluated the performance and updates the locomotory gaits to maximize the expected long‐term rewards. A comprehensive description of the swimming model training and performance could be found in our previous work.^[^ ^39^ ^]^ In the second learning session, a Soft Actor‐Critic (SAC) algorithm was implemented to enable the swimmer to identify a target direction based on local chemical cues detected by the chemical sensor.^[^ ^57^, ^58^ ^]^ The SAC algorithm leverages the actor‐critic structure and the clipped double‐Q trick. In this setup, the actor network predicts an action, while the critic network evaluated the action using two Q‐networks and two target Q‐networks. The target direction predicted by the SAC algorithm was then integrated with the trained PPO predictor to generate effective chemotactic navigation strategies. It was noted that the swimmer's configuration did not specify a head or tail. Therefore, for a given target direction, the swimmer could either rotate its head or its tail to align itself with the target, choosing the option that required the smaller angle of turn. However, due to intrinsic asymmetry in the training results, for a large angle turn where both rotations of head or tail were equally effective (e.g., 90° turn), the model exhibited a weak bias toward counterclockwise rotation. This asymmetry in selecting binary policies was common for reinforcement learning and had been observed in other models.^[^ ^59^, ^60^ ^]^ Nevertheless, these results suggest that this small bias in rotation handedness did not influence the effectiveness in navigation.

#### 2.2.2 Bio‐Inspired Sensing Mechanism

To achieve mapless navigation, a sensing mechanism inspired by biological microswimmers was proposed. In this mechanism, the swimmer senses the local chemical signal at a given time *C*(*t*_*n*_) and utilized short‐term memory to detect changes in chemical signal Δ*C* = *C*(*t*_*n* + 1_) − *C*(*t*_*n*_) relative to the change in the swimmer location Δ**r**_*c*_ and the current swimmer orientation θ_*c*_. While the mechanism based on detected chemical gradients might also be possible, this method would require additional definitions of state variables for the chemical gradients, which increase the computational cost of training. Thus, a simple temporal change was used in the chemical signal as the state variable, as it was sufficient for discriminating changes in chemical fields to determine the next action. While a recognition of the map was not required for mapless navigation, the swimmer would require a memory of the relative change in its location and orientation over times. This enables the swimmer to construct an “imaginary” map with the history of relative location and orientation during its navigation. Here, the duration was set between each signal detection to be *t*_*n* + 1_ − *t*_*n*_ = 50Δ*t*_ℓ_, where Δ*t*_ℓ_ = 0.1 is the time step for locomotory gait selection by the PPO predictor. Employing different timescales for gait selection and signal detection allowed the swimmer to obtain a more stable target direction, reducing the influence of small fluctuations in sensor locations due to rapid transitions between different locomotory gaits. The state space was thus defined as *s*_*t*_ ∈ (Δ**r**_*c*_, Δ*C*, θ_*c*_).

Here, the chemical signal is collected by a sensor placed at the centroid of the swimmer *C*(*t*) = *C*(**r**_*c*_) was assumed, but the RL framework could be generalized to sensors placed at other locations, where different sensor placements did not have significant influence in the navigation strategies (see a detailed discussion about the effects of sensor location in Section S3, Supporting Information). Based on the information given by *s*_*t*_, a new target direction was generated as an action *a*_*t*_ ∈ (θ_*T*_). The reward was set as *r*_*t*_ = Δ*C* × α|Δ**r**_*c*_|, accounting for both the swimmer's displacement and the temporal change in chemical signals. A value α = 100 was chosen to scale the reward to the order of 1, avoiding small values that may reduce the efficiency of the SAC algorithm.^[^ ^57^ ^]^ While other reward functions might also be possible, here the arguably simplest reward function for effective navigation was selected. More detailed discussions for the choices of reward function and hyperparameters are provided in Section S9 and S10 (Supporting Information).

The choice of different algorithms for training the predictor for locomotory gaits (PPO) and chemotactic navigation (SAC) strategies to optimize performance was remarked upon. PPO was an efficient algorithm with fast convergence for locomotion problems that could provide sufficient training data. However, PPO was less sample‐efficient compared to SAC, which was crucial for chemotactic navigation problems that necessitate a long history of data to evaluate the overall success of the developed policy. Therefore, SAC was used for its superior sample efficiency, leading to more stable training results for chemotactic navigation. Taken together, the two‐step learning scheme addresses the sampling challenge by employing RL algorithms tailored to each aspect of training: PPO for locomotion and SAC for navigation.

## 3 Results and Discussion

### 3.1 The “Run‐and‐Tumble” Chemotactic Behavior

**Figure 3:**
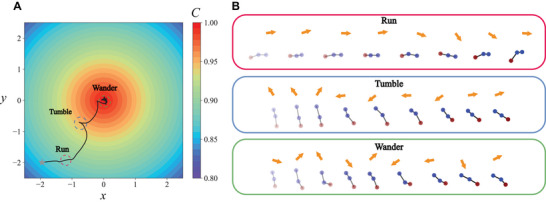
The mapless swimmer exhibits a biomimetic run‐and‐tumble strategy to navigate towards the chemical source. A) The swimmer switches between three distinct modes of run, tumble and wander to swim towards and stay in the region of chemical field with a maximum concentration. The color of the map denotes the local strength of the chemical field *C*. B) Gait schematics of the run, tumble, and wander modes. The time interval between consecutive gaits in the schematic is given by Δ*t*_*gait*_ = 50Δ*t*_ℓ_ = 5. In the run mode (red box), the swimmer tends to swim in a straight path with a small change in orientation. In the tumble mode (blue box), the swimmer performs rapid reorientation to correct its path when the path deviates from the desirable positive chemical gradient. In the wander mode (green box), the microswimmer stays at the location of maximum chemical concentration by selecting gaits with a non‐swimming actuation.

First, we examine the chemotactic behavior of a mapless swimmer immersed in a radially linear chemical field given by $C\left(x,y\right)=-\sqrt{x^{2}+y^{2}}/20+1$ (**Figure** **3**). Interestingly, the swimmer exhibits a chemotactic navigation strategy reminiscent of the run‐and‐tumble behavior observed in swimming bacteria (Figure 3A; Video S1, Supporting Information).^[^ ^24^, ^47^, ^61^ ^]^ Initially, the swimmer enters a “run” mode, moving along a positive chemical gradient with an approximately constant θ_*T*_ advised by the SAC agent (Figure 3B, red box). This mode features a smooth swimming trajectory with gradual steering to correct its path toward the chemical source. However, the swimmer may misinterpret θ_*T*_ due to limited localized chemical signals, causing it to deviate into an undesired path with a negative chemical gradient. When this occurs, the swimmer switches to a “tumble” mode to rapidly steer away from the unfavorable path and reorient itself towards a more favorable direction (Figure 3B, blue box). This run‐and‐tumble strategy enables the swimmer to successfully reach the chemical source, despite its location being unknown a priori. Once near the chemical source, the swimmer adopts a *“wander”* mode (Figure 3B, green box), frequently changing θ_*T*_ and performing gaits that results in minimal net swimming motion, a behavior also akin to what is observed in bacterial chemotaxis. This first example demonstrates how an artificial microswimmer develops a chemotactic strategy similar to its biological counterparts to navigate a chemical environment without relying on a pre‐existing map or prior knowledge of the environment.

**Figure 4:**
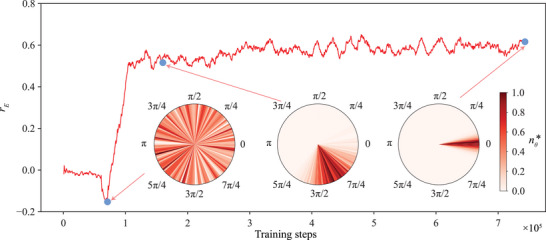
Model improvement throughout the training process of the mapless swimmer. ${\bar{r}}_{E}$ denotes the mean episodic training reward over previous 100 episodes. We extract checkpoint models at several representative points to evaluate the progress of the training process. The swimmer is set at the left side of the chemical source initially during evaluation. We use the results at *t* = 100 to obtain the action prediction from each model. 1000 predictions are generated for each case. The insets represent the distributions of the action predictions at different representative steps during the training process.

In **Figure** **4**, we examine the model's improvement throughout the training process for the mapless navigation. Models are periodically extracted and saved during training. We select three checkpoint models and visualize their predictions for the target direction θ_*T*_. In each scenario, we consider a swimmer initially positioned at **r**_*c*_ = [− 3, 0] with *L*_1_, *L*_2_ = 1 and θ_*A*_ = π. For each checkpoint model, we generate 1000 predictions by evaluating their θ_*T*_ at *t* = 100. These predictions are then used to create a normalized radial histogram, as depicted in Figure 4. The color density represents the normalized intensity $n_{\theta }^{*}=\left(n-n_{min}\right)/\left(n_{max}-n_{min}\right)$. Here, *n* is the original intensity at a certain range of angles, with *n*_*min*_ and *n*_*max*_ denoting the minimum and maximum intensity, respectively. At the beginning of the training, the model lacks any guidance for navigation, resulting in random predictions (Figure 4, left inset). As training progresses, these random predictions begin to converge (Figure 4, middle inset). After sufficient training, the model accurately predicts θ_*T*_ to effectively reach the target (Figure 4, right inset).

**Figure 5:**
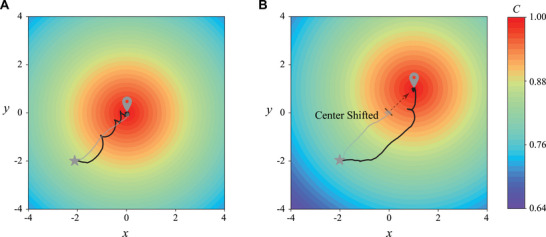
Comparison of the navigation performance of the mapless swimmer and the map‐based swimmer in the original training map and the shifted map. The blue and grey colored lines correspond to the trajectories of the mapless swimmer and map‐based swimmer, respectively. a) Both swimmers can search for the source effectively in the original training map. b) The mapless swimmer reaches the chemical source successfully in the shifted map, while the map‐based swimmer completely fails.

To illustrate directly how a mapless swimmer navigates differently from a map‐based swimmer (See Section S4, Supporting Information), we consider a simple scenario where the location of a chemical source is shifted. That is, we shift the location of the chemical source in the original training map from [0,0] to [1,1]. Yet, even for such a simple shift in chemical field, the map‐based swimmer will fail completely and never reach the source, as it cannot recognize the shift of the chemical source based on its training map. In contrast, the mapless swimmer does not rely on the training map for navigation and successfully navigates to the new chemical source location with a 100% success rate in 200 trials (**Figure** **5**A,B). The time taken for the swimmer to reach the chemical source has no significant difference before the shift (368.03 ± 9.42, MEAN±SEM) and after the shift (369.75 ± 10.8, MEAN±SEM). We note that a map‐based swimmer only exhibits the *“run”* mode to reach the chemical source directly, but this strategy is not robust to any small changes in the environment as demonstrated in the shifted map. The mapless swimmer exhibits the *“run”* mode to ascent the chemical gradient, and employs the *“tumble”* mode to redirect its path upon any deviation from the ascending chemical gradient. Finally, the *“wander”* mode enables the swimmer to stay in the maximum chemical field when the swimmer reaches the target. Thus, the mapless swimmer achieves more robust navigation based on the three navigation modes. This result underscores the importance of mapless navigation in dynamic or unpredictable environments.

We acknowledged that our mapless swimmer relies on local gradient sensing–an inductive bias that improves robustness and reduces training cost. It is worth noting that map‐based approaches, such as those using recurrent neural networks with a sufficiently large amount of data, may also achieve strong generalization.^[^ ^62^ ^]^ The selection between mapless and map‐based strategies should therefore consider the intended application, data availability, and computational resources.

### 3.2 Robustness of Chemotactic Navigation In Unknown Environments

**Figure 6:**
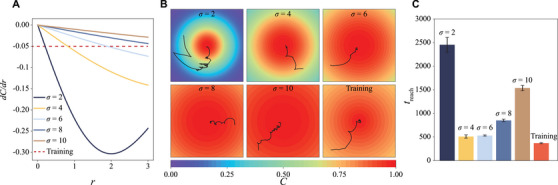
We systematically investigate the robustness of the mapless swimmer in navigating unknown environments with gradients different from the training environment. We consider chemical fields with normal distributions of different standard deviation σ. a) The solid lines depict the concentration gradients in the radial direction *dC*/*dr* for chemical fields with different σ. The constant dashed line represents the gradient of the training map with a radially linear distribution. b) Example navigation trajectories for the different chemical fields: i) σ = 2, ii) σ = 4, iii) σ = 6, iv) σ = 8, v) σ = 10 and vi) the training map. c) Comparison of the reaching time *t*_*reach*_ for different chemical fields. Total of 200 evaluations are performed for each case. The chemical gradient of the fields with σ = 4, 6, 8 has values close to that of the training map, so these three parameters have a relatively smaller *t*_*reach*_ compared to σ = 2, 10. We note that the case for σ = 2 has 41 fail samples out of 200 evaluation samples. In those failing cases, we count *t*_*reach*_ to be 6000 (the evaluation time limit). Therefore, we remark that the large *t*_*reach*_ for σ = 2 is still an underestimated value. The error bars represent the standard error of the mean.

The chemical fields considered in previous examples feature uniform radial concentration gradients (*dC*/*dr* = constant), similar to those in the training environment. Here, we probe further the robustness of the navigation strategy in chemical fields with non‐uniform concentration gradients, distinct from the training environment. To this end, we utilize a family of chemical fields described by the normal distribution, $C\left(x,y\right)=e^{-\frac{1}{2}\left[{\left(x/\sigma \right)}^{2}+{\left(y/\sigma \right)}^{2}\right]}$, where σ denotes the standard deviations. **Figure** **6**A contrasts the non‐uniform concentration gradients for different values of σ with the constant gradient employed in the training environment. In Figure 6B, we display the swimming trajectories of a mapless swimmer navigating these environments with non‐uniform chemical gradients.

We classify the swimming trajectories into three categories based on how closely the spatially averaged value of the non‐uniform chemical gradient matches the constant value (*dC*/*dr* = −0.05) used in training. The first group includes chemical gradients with spatially averaged values similar to the constant gradient from the training environment (e.g., σ = 4 and 6). In this group, the mapless swimmer displays qualitatively similar swimming trajectories to those observed in the training environment – they reach the chemical source with only a few tumbling events, resulting in comparable reaching times (Figure 6C). The second group consists of larger values of σ (e.g., σ = 8 and 10), where the mapless swimmer requires more tumbling events to correct its path, resulting in noisier trajectories (Figure 6B) and longer reaching times to navigate to the chemical source (Figure 6C). In all cases, we set a maximum simulation time of *t*_*max*_ = 6000, and the mapless swimmer successfully navigates towards the chemical source within this time frame in all 200 trials. Lastly, the non‐uniform chemical gradient with σ = 2 deviates significantly from the training environment. In this extreme case, despite a very noisy trajectory and substantially longer reaching time, the mapless swimmer is still able to navigate towards the chemical source with an approximately 80% success rate within the maximum simulation time. In general, the mapless swimmer is effective in navigating chemical fields with increasing gradients. The closer the gradients of the chemical fields to the linear gradient in the training model, the better the navigation performance is expected to be. Yet, the steeply increasing gradient for σ = 2 leads to significantly larger changes in detected chemical signals upon any motion, in contrast to the steady signals detected in the linear gradient of the training model, hence leading to counterproductive navigation. A better navigation performance in such steeply increasing gradient can possibly be achieved via re‐training the model in a steeper chemical gradient, but this may lead to reduced performance in other cases. Nevertheless, these results demonstrate the robustness of the mapless swimmer's chemotactic navigation capability in chemical fields spanning a wide range of gradients.

**Figure 7:**
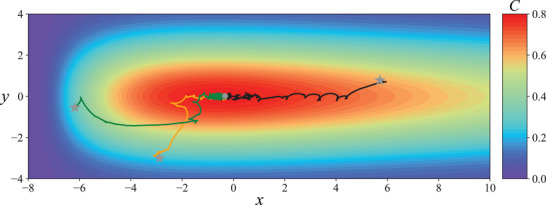
In a skewed chemical field with largely varying chemical gradients at different locations, the mapless swimmer can adaptively switches between the run mode and the tumble mode in accordance with the localized chemical gradients. This enables the swimmer to select different paths to achieve gradient ascent effectively in an environment with an unpredictable change in gradient.

In addition to non‐uniform radial concentration gradients, we introduce asymmetry to the chemical gradients by considering a skewed distribution described by a log‐normal distribution along the *x*‐direction, $C\left(x,y\right)=\frac{5}{\pi \sigma^{2}}e^{-\frac{1}{2}\left[{\left(\frac{\ln(x+8)}{\sigma }\right)}^{2}+{\left(\frac{y}{\sigma }\right)}^{2}\right]}$. **Figure** **7** displays the chemotactic behavior of the mapless swimmer in such a skewed chemical field (see also Video S2, Supporting Information). Three representative trajectories from initial positions with gradients of varying steepness towards the chemical source are illustrated in Figure 7. The mapless swimmer uses different locomotory gaits to navigate these diverse gradients. The swimmer on the left (green) and the swimmer at the bottom (orange) of the map choose paths with mild gradient increases and predominantly use the run mode. As they approach the region near the chemical source with a steep gradient, they frequently switch between run and tumble modes to locate the source precisely. The swimmer on the right (black) initially encounters a relatively mild gradient along its path to the source, selecting a relatively straight path but needing to tumble frequently to correct its course. These examples demonstrate that the mapless swimmer can adaptively switch between run and tumble modes to choose different paths for gradient ascent.

### 3.3 Navigating A Complex Chemical Field With Multiple Local Maxima

**Figure 8:**
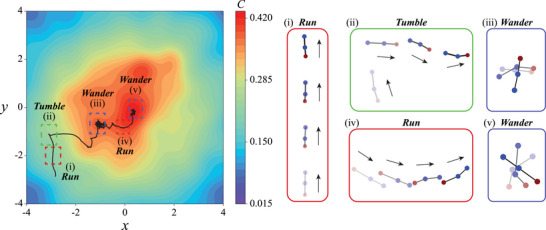
The mapless swimmer can progressively explore its surrounding to navigate in a complex chemical field with multiple local maxima. The swimmer first reaches a local maxima with a run‐and‐tumble strategy. It then progressively explores and reaches the other local maxima with a higher concentration. The gait schematics highlighted by the red, green and purples boxes show some representative snapshots of the (i), (iv) run, (ii) tumble and (iii), (v) wander modes occurring at different stages of the swimmer navigation, respectively.

Moreover, we showcase the chemotaxis of a mapless swimmer in a complex chemical field characterized by multiple local maxima in concentration, as depicted in **Figure** **8** (see Section S6, Supporting Information for the construction details of this irregular chemical field). Initially, the swimmer employs a run‐and‐tumble strategy, similar to previous examples, to perform gradient ascent (Figure 8i,ii), successfully navigating toward a local concentration maximum (Figure 8 iii). Upon reaching this local maximum, the swimmer switches to the *“wander”* mode, performing a localized search (Figure 8 iii). The stochastic nature of the wander mode enables the swimmer to continually sample chemical gradients around the local maximum, thereby facilitating the exploration of other maxima with potentially higher concentrations and preventing entrapment at a single local maximum. After escaping from the initial local maximum, the swimmer returns to the run‐and‐tumble strategy (Figure 8 iv), navigating towards another local maximum (Figure 8v), which in this case has a higher concentration. This example illustrates an intriguing chemotactic behavior characterized by progressive exploration toward the global concentration maximum. However, it is important to note that the mapless swimmer is not guaranteed to reach the global maximum under all conditions, as reinforcement learning often targets local maxima.^[^ ^63^, ^64^ ^]^ For multiple local maxima that are well separated in space, the swimmer can get trapped in one of the local maxima (See Section S5, Supporting Information). Nevertheless, the ability of the mapless swimmer to stochastically explore around local maxima opens the possibility of eventually reaching the global maximum in complex chemical fields, as demonstrated in this example.

### 3.4 Chemotaxis in Time‐Varying Fluctuating Environment

**Figure 9:**
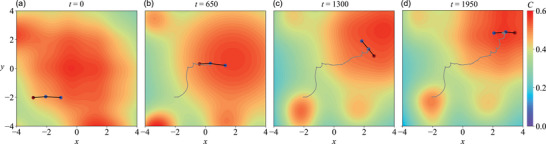
The microswimmer is capable of navigating in a time‐varying fluctuating chemical field. The chemical field has its global maxima initially located at the center, and is gradually changing its shape as well as the global maxima position to the right top corner. The swimmer can adapt to the change in chemical field and navigate toward the global maxima. The snapshots show the evolution of the chemical field and the swimmer's position at different time steps, and the gray lines represents the trajectories of the swimmer: a) *t* = 0, b) *t* = 650, c) *t* = 1300, and d) *t* = 1950.

Finally, we demonstrate the capability of the mapless swimmer in navigating a time‐varying, fluctuating chemical field, as shown in **Figure** **9** (see Section S7, Supporting Information for the construction details of this fluctuating chemical field). This fluctuating chemical field consists of a global maxima and smaller peaks which stretch radially and moves over time (Figure 9A–D; Video S4, Supporting Information), hence resulting in a complex, dynamic environments relevant to many biological media with unpredictable noises. Nevertheless, the mapless swimmer can still adopt run‐and‐tumble strategy as previous examples to navigate toward the global maximum (Figure 9A,B) and dynamically tracing it (Figure 9C,D). We tested several dynamical fields with random temporal variations in global maxima and small peaks as described in Section S7 (Supporting Information), and the swimmer can achieve successful navigation in all cases (results not shown in here for brevity). Overall, all examples in Figures 6, 7, 8, 9 highlight the robustness of the RL‐advised chemotaxis strategies employed by the mapless swimmer, demonstrating its effectiveness in navigating in various complex chemical fields similar to biological media, irrespective to whether the environment is static or dynamic.

## 4 Conclusion 

In this work, we explored the chemotactic behavior of artificial microswimmers utilizing a mapless navigation strategy powered by deep RL. Our findings highlight several key advancements in the development of fully autonomous artificial microswimmers capable of performing complex navigation tasks without relying on pre‐existing maps or external guidance. Firstly, we demonstrated that a mapless swimmer, equipped with a deep RL‐based controller, can effectively perform chemotaxis by adapting its locomotory gaits in response to local chemical signals. The swimmer exhibited a run‐and‐tumble strategy, similar to the behavior observed in bacterial chemotaxis, enabling it to navigate towards chemical sources in various complex environments.

In contrast to a map‐based swimmer that relies on prior knowledge of the target location, the mapless swimmer exhibited remarkable robustness in navigating chemical fields that significantly deviated from the training environment. This robustness was evident in the swimmer's ability to navigate through non‐uniform and skewed chemical gradients, fields with multiple local maxima, as well as fluctuating fields with unpredictable noises. The swimmer's stochastic wander mode allowed it to explore around local maxima, enhancing the likelihood of finding global maxima and preventing entrapment. Taken together, these results underscore the significant potential of the deep RL approach in developing generalizable and robust navigation strategies for artificial microswimmers in complex environments. This opens up new possibilities for biomedical applications, such as targeted drug delivery and microsurgery, where autonomous operation in dynamic and unstructured environments is crucial.

Finally, we remark that although this study focuses on the navigation of a model three‐sphere swimmer in chemical fields, the deep RL approach can be readily generalized to mapless navigation problems based on other environmental cues^[^ ^34^, ^65^, ^66^, ^67^ ^]^ and to other reconfigurable swimmer designs.^[^ ^68^, ^69^, ^70^, ^71^, ^72^, ^73^ ^]^ Future research will investigate the tactic behavior of microswimmers under other stimuli, such as thermal or electric fields. More advanced computational methods can be implemented for navigation problems in more complex environments with obstacles and irregular boundaries.^[^ ^74^ ^]^ As a closing remark, we highlight that the integration of sensing, memory, and neural network into sub‐millimeter swimmers remains a significant experimental challenge.^[^ ^38^ ^]^ Recent advancement on microrobots with on‐board sensors and actuators may provide potential solutions for bridging in silico autonomy and real‐world navigation through minimal policy networks and novel neuromorphic hardware.^[^ ^75^, ^76^ ^]^ We call for experimental validation of the proposed strategies to translate these findings into practical applications.

## Conflict of interest

The authors declare no potential conflict of interests.

## Author Contributions

Y.L., O.S.P., and A.C.H.T performed research; Y.L., O.S.P., and A.C.H.T analyzed data; and Y.L., O.S.P., and A.C.H.T wrote the paper.

## Supporting information

Supporting Information

Supplemental Video 1

Supplemental Video 2

Supplemental Video 3

Supplemental Video 4

## Data Availability

The data that support the findings of this study are available from the corresponding author upon reasonable request.
