# Supplementary material for: Learning to Navigate in Chemical Fields Without A Map at Low Reynolds Numbers
Source: Adv Sci (Weinh). 2025 Aug 28;12(41):e10092. doi: 10.1002/advs.202510092 (PMC12591185; doi:10.1002/advs.202510092)
Supplement: Supplementary file 1 — Supporting Information [file ADVS-12-e10092-s004.pdf]

Supplemental Materials for "Learning to navigate in chemical fields without a map at low Reynolds numbers"

Yangzhe Liu[1], On Shun Pak[2] , Alan C. H. Tsang[1]*

[1]Department of Mechanical Engineering, University of Hong Kong, Hong Kong, China
[2]Department of Mechanical Engineering, Santa Clara University, Santa Clara, CA 95053, USA

Corresponding authors: * alancht@hku.hk

1 Description of supplemental movies

Movie S1: Supplementary video for Fig. 3. A mapless swimmer exhibits a biomimetic run-and-tumble navigation strategy to search for the chemical source. The red arrow indicates the instantaneous target direction of the mapless swimmer.

Movie S2: Supplementary video for Fig. 7. Overlay of three individually simulated sample trajectories for the mapless swimmer navigating in a skewed chemical field with a log-normal concentration distribution.

Movie S3: Supplementary video for Fig. 8. A mapless swimmer performs progressive exploration in complex chemical field with multiple local maxima.

Movie S4: Supplementary video for Fig. 9. A mapless swimmer adopt run-and-tumble strategy to navigate in time-varying, fluctuating chemical field and dynamically tracing the global maximum.

2 Non-dimensionlization

In this section, we briefly introduce the non-dimensionization of all the parameters in our calculation. We scale all the length by the maximum link length L_{max}, and velocities by a characteristic link actuation velocity V_c, then the resultant time is $T = L_{max}/V_c$. Moreover, the force and torque are scaled by $\mu V_c L_{max}$ and $\mu V_c L_{max}^2$ respectively. Thus, the dimensionless link length of the microswimmer ranges from $L^*_{min} = 0.6$ to $L^*_{max} = 1$, the dimensionless link actuation velocity ranges from $V^*_{min} = -4$ to $V^*_{max} = 4$, and the dimensionless actuation rate for the between two links ranges from $\theta^*_{min} = -2\pi/3$ to $\theta^*_{max} = 2\pi/3$. The dimensionless time duration for locomotory gait selection by PPO is then set to $\Delta t^*_l = 0.1$.

3 Effects of different sensor placements

Here we investigate how the different sensor placements influence the navigation performance of the mapless microswimmer. In the main text, we consider the swimmer which uses the centroid as the sensory location for detection of local chemical signals. Here we consider cases with sensors placing on other locations of the swimmer: sensor placing on a sphere at the head of the swimmer, sensor placing in the middle sphere, and sensors placing in all spheres. We first

set up the state for each placement plan as below:

$$\begin{aligned} s_{head} &\in (\Delta\mathbf{r}_1, \Delta C(\mathbf{r}_1), \theta_c) \\ s_{mid} &\in (\Delta\mathbf{r}_2, \Delta C(\mathbf{r}_2), \theta_c) \\ s_{all} &\in (\Delta\mathbf{r}_c, \Delta\overline{C}(\mathbf{r}_c), \theta_c) \end{aligned} \tag{S1}$$

Here $\Delta\overline{C}(\mathbf{r}_c) = [\Delta C(\mathbf{r}_1) + \Delta C(\mathbf{r}_2) + \Delta C(\mathbf{r}_3)]/3$, which denotes the averaged change in chemical signals detected by the three spheres. The action remains the same as the case in the main text: $a_t \in (\theta_T)$. The reward for each case is then defined as:

$$\begin{aligned} r_{first} &= \Delta C(\mathbf{r}_1) \times \alpha|\Delta\mathbf{r}_1| \\ r_{mid} &= \Delta C(\mathbf{r}_2) \times \alpha|\Delta\mathbf{r}_2| \\ r_{all} &= \Delta\overline{C}(\mathbf{r}_c) \times \alpha|\Delta\mathbf{r}_c| \end{aligned} \tag{S2}$$

We choose the same scaling factor $\alpha = 100$ as the main text, and the same chemical field is used for the training (i.e., $C = -\sqrt{x^2 + y^2}/20 + 1$). The three sensor placement plans are trained separately, and we compare the navigation of the swimmer in each case.

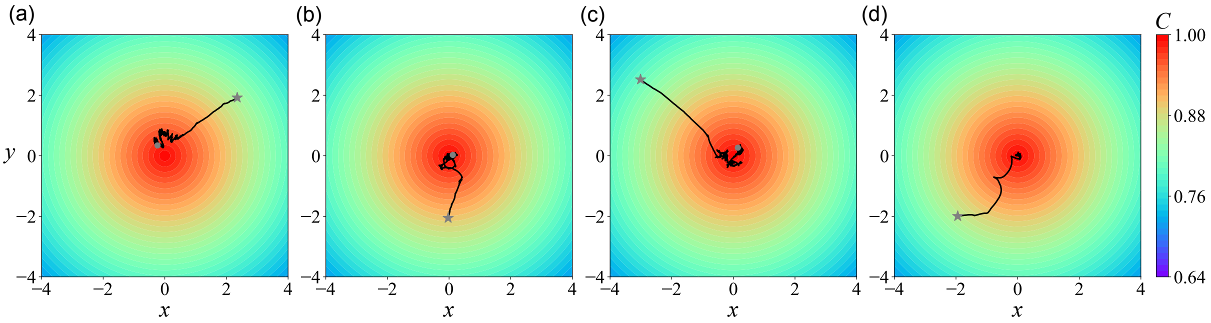

Figure S1: Sample trajectories for the centroid of the mapless swimmer with different sensor placements: (a) Sensor placed at the head/tail. (b) Sensor placed at the middle sphere. (c) Sensors placed at all three spheres. (d) Sensor place at the centroid (the same sensor placement as in the main text).

Sample trajectories for the mapless swimmer with different sensor placements are depicted in Fig. S1. For the swimmer with its sensor placed at the head, the swimmer exhibits similar run-and-tumble strategies as the swimmer with the centroid sensor in the main text [Fig. S1(a) vs. Fig. S1(d)]. Since the sensor is placed at the head, the swimmer will reach the chemical source with its head staying at the target location. The centroid of the swimmer will not reach the source in this case. Also, the average reaching time for the swimmer's head to reach to chemical source ($t_{head} = 271.4 \pm 8.4$, mean $\pm$ sem, $n = 200$ simulations) is smaller than the reaching time for the swimmer in the main text which reaches the source with its centroid ($t_{centroid} = 369.8 \pm 10.8$, mean $\pm$ sem, $n = 200$ simulations). Since both swimmers do not reach the chemical source with the same reference (centroid, the direct comparison between the navigation performance of both cases based on their reaching time is not relevant. For the swimmer with its sensor placed at the middle sphere, the swimmer has a relatively noisier trajectory and has a longer average reaching time ($t_{mid} = 975.2 \pm 47.8$, mean $\pm$ sem, $n = 200$ simulations), see Fig. S1(b) vs. Fig. S1(d). This is due to the fact that the middle sphere typically has a larger displacement compared to the centroid in each actuation, therefore the chemical signal received by the middle sphere also fluctuates more. For the swimmer with sensors placed at all spheres, the swimmer fails to stay at the location of the chemical source when it gets close to the source [Fig. S1(c)]. This is because the swimmer considers the average chemical signals of all spheres to determine its action, and the swimmer obtains too much unnecessary information. As a result, the swimmer cannot achieve accurate navigation to the target location.

We remark that all the above cases with different sensor placements can generate reasonable navigation strategies to guide the swimmer towards the chemical source. A better performance for the above models with the three alternative sensor placements can be achieved by further fine-tuning the training parameters. These results show that our reinforcement learning approach can be generalized to swimmers with different sensor designs.

4 Training of a map-based swimmer

In main text Fig. 5, we compare the navigation performance of the mapless swimmer and the map-based swimmer. Here we provide supplementary information about the training model of the map-based swimmer. The state space for a map-based swimmer is given by $s_t \in (\mathbf{r}_c, C)$ with the reward given by the instantaneous chemical signal $r_t = C$. The action is kept the same as the mapless navigation to generate a target direction $a_t \in (\theta_T)$. That is, the map-based swimmer does not use the difference in chemical fields over times as the state space but using the local chemical field strength directly. Therefore, its navigation performance is not robust to the change in the chemical field as shown in Fig. 5.

5 Evaluation on a chemical field with two peaks

In this section, we evaluate the navigation of the mapless swimmer in a chemical field with two peaks. The chemical field map is generated by adding two simplified bivariate normal distributions:

$$C_{2peak}(x,y) = \frac{\alpha_1}{2\pi\sigma^2} e^{-\frac{1}{2}[(\frac{x-\mu_{x1}}{\sigma})^2 + (\frac{y}{\sigma})^2]} + \frac{\alpha_2}{2\pi\sigma^2} e^{-\frac{1}{2}[(\frac{x-\mu_{x2}}{\sigma})^2 + (\frac{y}{\sigma})^2]}. \tag{S3}$$

Here, $\sigma = 2$ denotes the standard deviation for the distribution. The means are set to $\mu_{x1} = -3$ and $\mu_{x2} = 3$ to define the position of the maximum chemical field point. To achieve two peaks with different values, we set the scaling factor $\alpha_1 = 5$ and $\alpha_2 = 4$.

We then test our mapless navigation model by placing the swimmer inside the chemical field under two conditions: the initial starting point being slightly closer to the left peak and the initial starting point at the middle of the two peaks (the location where the chemical field is minimum). Our reinforcement learning algorithm is a stochastic policy, meaning that the swimmer follows a probability distribution to select which policies to implement. Therefore, different trials of simulations with the same initial conditions may still generate different results. We test each condition with 100 samples and evaluate the final positions. For the condition where the swimmer starts from the point near to the left peak, all the final positions fall into the left peak eventually [Fig. S2(a)]. For the condition where the swimmer starts from the middle point between two peaks, 45 samples reach the left peak, and 55 samples reach the right peak [Fig. S2(b)]. We note that slightly more samples fall on the right peak because the initial position of the swimmer is slightly closer. These results indicate that the mapless swimmer tends to swim towards the nearest local maxima of the chemical source.

6 Construct the irregular map

Here we briefly elaborate on how we construct the irregular map in section 3.3 of the main text. We first create a bivariate normal distribution with mean $\mu_x, \mu_y = 0, 0$ and covariance matrix $\mathbf{H} = [10, 0.5; 0.5, 10]$, and randomly sample 8500 data points. We then utilize a nonparametric estimation technique called kernel density estimation (KDE) on the sampled data to create a non-perfect estimator [1]. The bivariate kernel estimator can be expressed as:

$$\hat{f}_K(x,y) = \frac{1}{n}\sum_{i=1}^{n} K_{h_x}(x-x_i)K_{h_y}(y-y_i), \tag{S4}$$

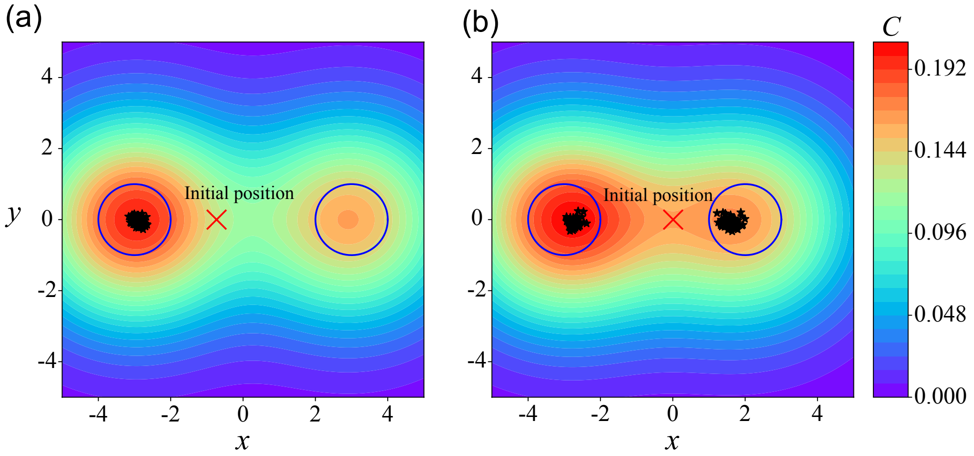

Figure S2: Evaluation result for the two-peak chemical field. (a) Final position of the microswimmer for the condition: Initial position is near the left peak. (b) Final position of the microswimmer for the condition: Initial position is at the middle between two peaks.

where K_h denotes the kernel function transformed by the bandwidth parameter h ($K_h(t) = K(t/h)/h$). We choose the Gaussian kernel as our kernel function and select the bandwidth $h = 0.13$ to perform an inaccurate estimation. Thus, the KDE then estimates a probability density function to generate a distribution with an irregular shape same as the one shown in main text Fig. 8.

7 Construct the map of time-varying fluctuating chemical field

In this section, we briefly illustrate how we construct the time-variant and dynamically changing chemical map. The chemical field map is constructed by superpositioning multiple perturbed bivariate normal distributions together, where each distribution can be expressed as the probability density function:

$$\begin{aligned} C(x, y, t) &= \frac{1}{2\pi\sigma_x\sigma_y\sqrt{1-\rho^2}} \exp\left[-\frac{Z}{2(1-\rho^2)}\right]\gamma_x(t)\gamma_y(t), \\ Z &\equiv \frac{(x-\mu_x(t))^2}{\sigma_x^2} - \frac{2\rho(x-\mu_x(t))(y-\mu_y(t))}{\sigma_x\sigma_y} + \frac{(y-\mu_y(t))^2}{\sigma_y^2}. \end{aligned} \tag{S5}$$

Here $\rho = V_{xy}(t)/(\sigma_x\sigma_y)$ and $V_{xy}(t)$ denotes the time dependent covariance, and the perturbation term γ can be expressed as:

$$\gamma_x(t) = 1 + 0.01\sin(f_x(t)\cdot x), \quad \gamma_y(t) = 1 + 0.01\cos(f_y(t)\cdot y). \tag{S6}$$

Here $f_x(t), f_y(t)$ are the time dependent perturbation frequencies. $f_x(t)$ is set to increase linearly from -3 to 3 from $t = 0$ to $t = 1500$, and stays at 3 afterwards, with $f_y(t)$ varying oppositely with $f_x(t)$. The entire chemical map can be constructed by adding multiple perturbed bivariant normal distribution together: $C_{dynamic} = \sum_{i=1}^{9} C_i(x, y, t)$.

8 Algorithm Pseudocode

In this section, we show the pseudocode of the SAC algorithm we use in the main text:

Algorithm 1 Soft Actor-Critic

Input: Initial policy parameters θ. Initial Q function parameter ϕ_1, ϕ_2. Initialize empty replay buffer $\mathcal{D}$
Initialize target Q function: $\phi_{target,1} \leftarrow \phi_1, \phi_{target,2} \leftarrow \phi_2$
for $k = 1, 2, 3...K$ **do**
  With current state s, select action $a \sim \pi_\theta(\cdot|s)$
  Observe new state s', reward r, and termination signal d
  Append the transition (s, a, r, s', d) to replay buffer $\mathcal{D}$
  if Update **then**
    Random sample N batches of transition $B = \{(s, a, r, s', d_i)_N\}$ from replay buffer $\mathcal{D}$
    Compute targets for Q functions with action $\tilde{a} \sim \pi_\theta(\cdot|s')$:
    $y(r, s', d) = r + \gamma(1-d)[\min_{j=1,2} Q_{\phi_{target,j}}(s', \tilde{a}') - \alpha \log \pi_\theta(\tilde{a}'|s')]$, for $j = 1, 2$
    Update Q functions using:
    $\nabla_{\phi_j} \frac{1}{|B|} \sum_{(s,a,r,s',d) \in B} (Q_{\phi_j}(s, a) - y(r, s', d))^2$, for $j = 1, 2$
    Use reparametrization trick to sample action $\tilde{a}_\theta(s)$, and update policy using:
    $\nabla_\theta \frac{1}{|B|} \sum_{s \in B} (\min_{j=1,2} Q_{\phi_j}(s, \tilde{a}_\theta(s)) - \alpha \log \pi_\theta(\tilde{a}_\theta(s)|s))$
    Update the temperature α using:
    $\nabla_\alpha \frac{1}{|B|} \sum_{s \in B} (-\alpha \log \pi_\theta(a|s) - \alpha \mathcal{H}_0)$
    Update target network weight: $\phi_{target,j} \leftarrow \tau \phi_{target,j} + (1-\tau)\phi_j$,for $j = 1, 2$
  end if
end for

9 Choice of hyperparameters

Name of Hyperparameter	Value (map-based)	Value (mapless)	Description
Learning rate	0.00015	0.00015	The learning rate used by Gradient descent
Target network update frequency	2	2	Number of gradient steps to run for each target network update
Neural network architecture	128,128	128,128	Size of two hidden layers for the policy and Q networks
Batch size	500	500	Number of training cases to be computed at each gradient update
Buffer size	1000000	1000000	Size of the replay buffer
Discount factor	0.99	0.99	Factor for computing discounted future reward
Activation function	ReLU	ReLU	Activation function

Table S1: Hyperparameters for deep reinforcement learning.

10 Effects of hyperparameters and reward function

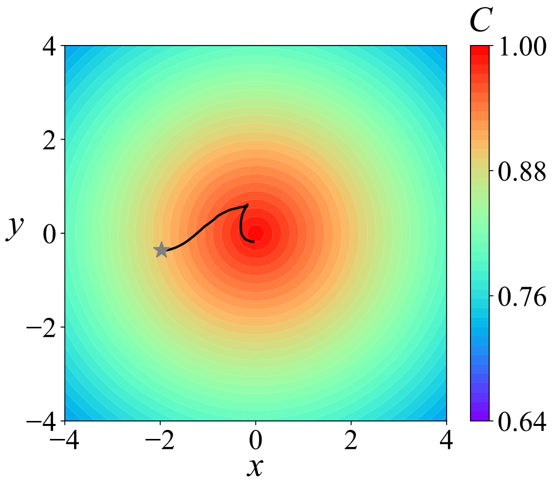

Figure S3: Sample trajectory for different hyperparameter settings also leads to a similar run-and-tumble strategy. We retrain a different navigation model with different hyperparameter settings, but keep all the other settings the same (state, action and reward).

In this section, we investigate the effects of hyperparameters and reward functions on the training results. We first quantitatively compare the navigation performance of models with other hyperparameter settings with the baseline model we used in the main text and listed in Supplementary Section 9. We strategically consider a testing model which the hyperparameters are expected to be less efficient compared to the baseline model (i.e., smaller learning rate, smaller update frequency, smaller neural network and smaller discount factor) and see how such an inefficient model performs. We adjust the learning rate from 0.00015 of the baseline model to 0.0003, target network update frequency from 2 to 1, neural network size from [128,128] to [64,64] and a discount factor from 0.99 to 0.997. The new model presents demonstrates a similar run-and-tumble strategy (Fig. S3) and achieves a 100% success rate in navigation across

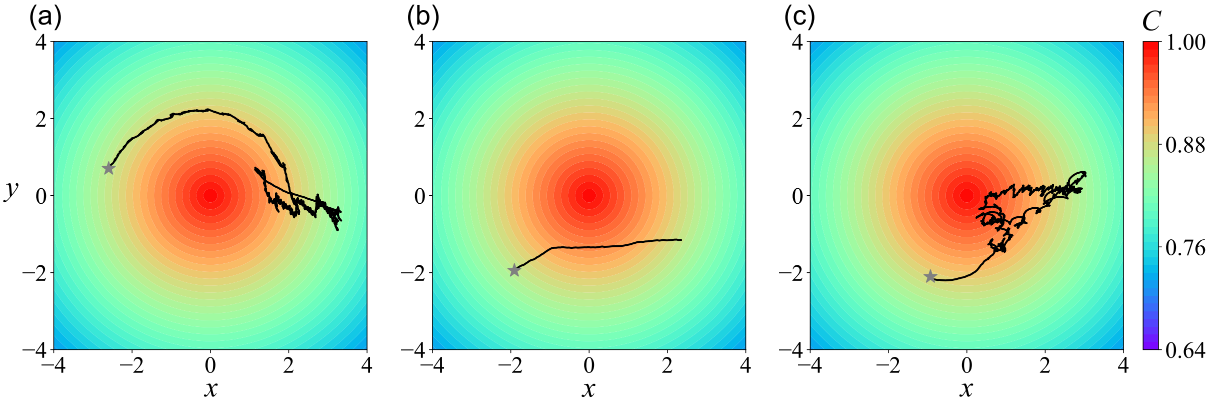

Figure S4: Sample trajectories for different reward function settings. (a) The sample simulation for reward function setting $r_t = \Delta C$, swimmers sometimes fail to search and redirect to the global maximum chemical field point (60% successful rate). (b) The case for reward function setting $r_t = |\Delta \mathbf{r}_c|$, swimmers is unable to sense the chemical field changes, thus swimmers only have 4.5% success rate to reach the target. (c) The case for $r_t = \Delta C_t + r_{t-1}$ implies the microswimmer can sense the cumulative chemical field changes; such condition have 75% success rate to reach the maximum chemical field source.

200 trials. We observe no observable differece in terms of chemotaxis strategies for reaching the target. Yet, the time taken for the swimmer to navigate to the maximum chemical field in this new model (522.47 ± 27.05, MEAN±SEM) is longer than the baseline model presented in the main text(368.03 ± 9.42, MEAN±SEM). Nevertheless, our results demonstrate that even if we consider a model with less efficient hyperparameters, our approach still demonstrates robust navigation performance and is able to reach the target with 100% success rate.

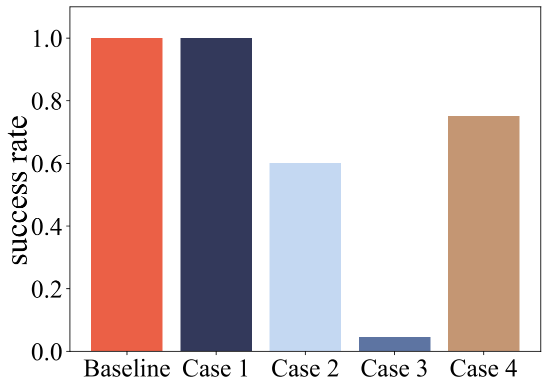

Figure S5: Success rate for different cases to reach the maximum chemical source within the limited time. Both the baseline model (mapless model in the main text) and case 1 (different hyperparameter condition) have 100% success rate to reach the target. Case 2 with simplified reward $r_t = \Delta C$ has 60% success rate, case 3 with reward $r_t = |\Delta \mathbf{r}_c|$ is almost unable to reach the target with only 4.5% success rate. The case 4 with the cumulative chemical field reward $r_t = \Delta C_t + r_{t-1}$ has 75% success rate to reach the target.

We then investigate how different reward functions influence the navigation performance, as shown in Fig. S4. Following the same evaluation setting as Fig. 6(c) in the main text, we set the maximum simulation time of $t_{max} = 6000$ and perform simulations over 200 samples for each reward settings. Recall that the reward function for the baseline model in the main text is defined as $r_t = \Delta C \times \alpha |\Delta \mathbf{r}_c|$, we now consider three cases with alternative reward functions: Fig. S4 (a) corresponds to the sample trajectory when the reward function is changed to $r_t = \Delta C$ (case 1), where the swimmer relies solely on sensing the chemical gradient. This setting results in significantly poorer performance, and the swimmer reaches the target in only 120 out of 200 evaluation samples (60% success rate) as shown in Fig. S5. The time required for the swimmer to reach the target in those 120 successful samples are 1605.08±156.96 (MEAN±SEM). Fig. S4 (b) corresponds to the sample trajectory when the reward function is changed to to $r_t = |\Delta \mathbf{r}_c|$, where

the swimmer cannot sense the change in chemical field and relies on randomness in swimming to reach the target. In this case, the performance deteriorates further, and the swimmer has only 9 successful samples out of 200 samples (4.5% success rate as shown in Fig.S5). This also implies that the swimmer is unable to perform effective chemotaxis without any chemical field information. Finally, Fig. S4 (c) corresponds to the sample trajectory as $r_t = \Delta C_t + r_{t-1}$, where the swimmer senses the cumulative chemical gradient over time. In this case, the model has 150 successful cases out of 200 evaluation samples (75% success rate in Fig. S5). The time required for the swimmer to reach the target is 1620.03 ± 119.52, MEAN±SEM. Therefore, we can conclude that the reward function settings indeed have a significant influence on the navigation performance and the reward function we have chosen for the baseline model is a simple and efficient choice for getting 100% success rate in navigation.

References

[1] Scott, D. W. Multivariate density estimation and visualization. *Handbook of computational statistics: Concepts and methods* 549–569 (2012).
